# Transmission Dynamics of Carbapenemase-Producing Klebsiella Pneumoniae and Anticipated Impact of Infection Control Strategies in a Surgical Unit

**DOI:** 10.1371/journal.pone.0041068

**Published:** 2012-07-31

**Authors:** Vana Sypsa, Mina Psichogiou, Georgia-Aikaterina Bouzala, Linos Hadjihannas, Angelos Hatzakis, Georgios L. Daikos

**Affiliations:** 1 Department of Hygiene, Epidemiology and Medical Statistics, Athens University Medical School, Athens, Greece; 2 1st Department of Internal Medicine-Propaedeutic, Laikon General Hospital, University of Athens, Athens, Greece; Université d'Auvergne Clermont 1, France

## Abstract

**Background:**

Carbapenemase-producing *Klebsiella pneumoniae* (CPKP) has been established as important nosocomial pathogen in many geographic regions. Transmission from patient to patient via the hands of healthcare workers is the main route of spread in the acute-care setting.

**Methodology/Principal Findings:**

Epidemiological and infection control data were recorded during a prospective observational study conducted in a surgical unit of a tertiary-care hospital in Greece. Surveillance culture for CPKP were obtained from all patients upon admission and weekly thereafter. The Ross-Macdonald model for vector-borne diseases was applied to obtain estimates for the basic reproduction number *R_0_* (average number of secondary cases per primary case in the absence of infection control) and assess the impact of infection control measures on CPKP containment in endemic and hyperendemic settings. Eighteen of 850 patients were colonized with CPKP on admission and 51 acquired CPKP during hospilazation. *R_0_* reached 2 and exceeded unity for long periods of time under the observed hand hygiene compliance (21%). The minimum hand hygiene compliance level necessary to control transmission was 50%. Reduction of 60% to 90% in colonized patients on admission, through active surveillance culture, contact precautions and isolation/cohorting, in combination with 60% compliance in hand hygiene would result in rapid decline in CPKP prevalence within 8–12 weeks. Antibiotics restrictions did not have a substantial benefit when an aggressive control strategy was implemented.

**Conclusions/Significance:**

Surveillance culture on admission and isolation/cohorting of colonized patients coupled with moderate hand hygiene compliance and contact precautions may lead to rapid control of CPKP in endemic and hyperendemic healthcare settings.

## Introduction

Over the past decade, carbapenem resistant *Klebsiella pneumoniae* is emerging as a major public health threat in many geographic areas [1]–[5]. This type of resistance is mediated by plasmid-borne β-lactamases (carbapenemases), mainly the serine-carbapenemase KPC and the metallo-β-lactamases VIM, IMP, and NDM [4], [6]. Once carbapenemase-producing *K. pneumoniae* (CPKP) are introduced into a health care facility with inadequate infection control practices, they may colonize a substantial number of patients and cause serious infections associated with adverse outcomes, prolonged hospital stay and increased costs [7]–[12].

An important step towards controlling CPKP is to gain insight on the mechanisms by which these organisms disseminate within a healthcare facility and estimate the extent to which different infection control measures may contribute to CPKP containment. In the past few years, mathematical modeling has been used to assess the impact of measures to control the spread of pathogens - such as Methicillin-resistant *Staphylococcus aureus* (MRSA) and Vancomycin-resistant *Enterococci* (VRE) - within the hospital setting and improve our ability to determine the quantitative effects of individual infection control measures [13]–[21]. An important contribution of these models is their ability to estimate not only the effectiveness of each infection control measure, but also the effectiveness of combinations of measures, and determine those most suited for the particular setting and pathogen [22], [23].

In the present study we applied a mathematical model on microbiological surveillance data for CPKP colonization/infection collected during a non-interventional study that was conducted in a tertiary care hospital located in Athens, Greece, an area with high prevalence of CPKP infections [24], [25]. Our aims were to provide estimates of CPKP transmissibility as well as to assess the impact of various infection control interventions on CPKP containment.

**Figure 1:**
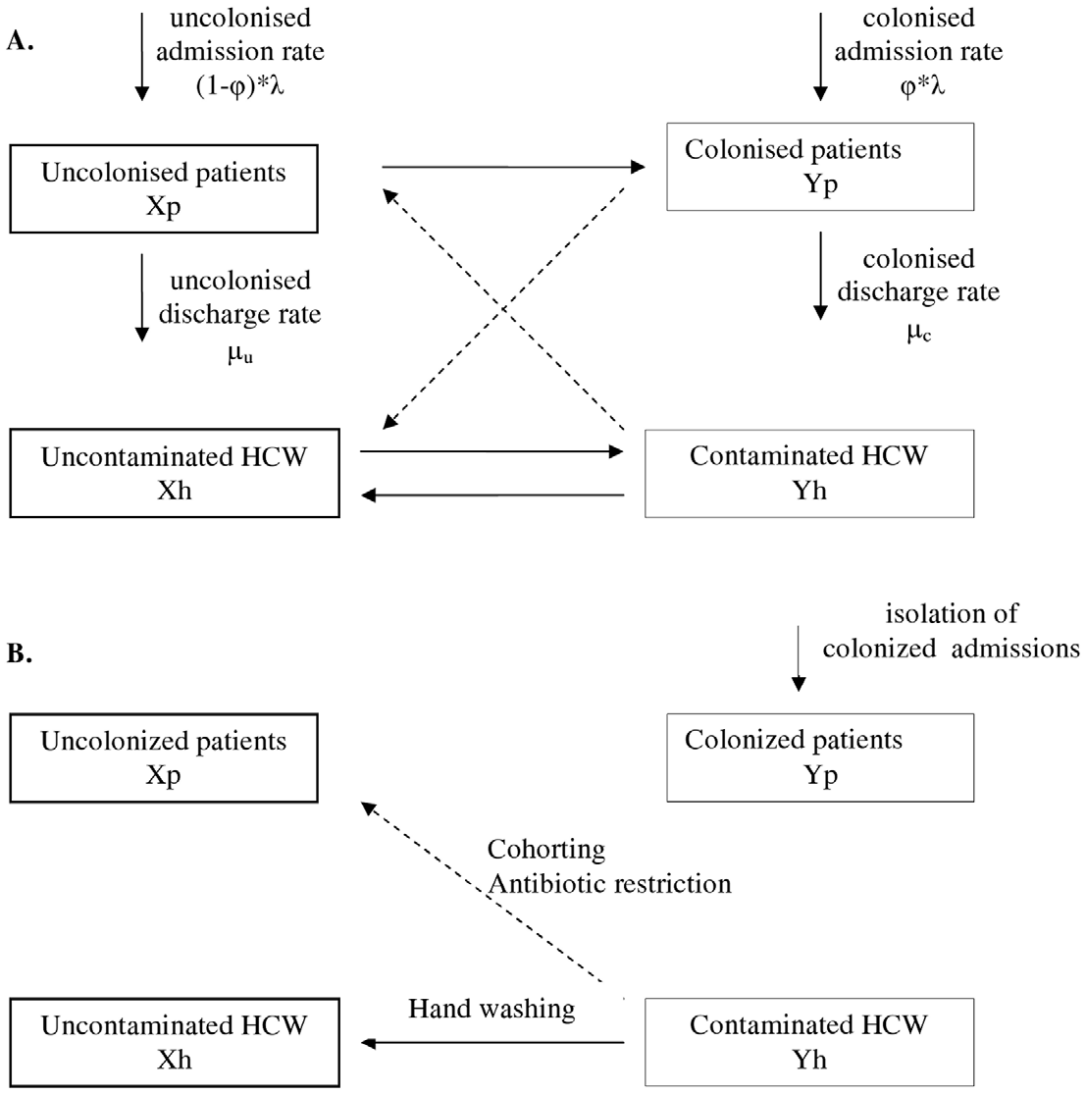
Model of indirect transmission of CPKP between patients through health-care workers (HCWs) and impact of intervention measures. A. Model of indirect transmission of CPKP between patients through health-care workers (HCWs) who act as vectors. Solid lines depict the movement to/from the four population groups and dashed lines depict the transmission between patients and HCWs B. The impact of intervention measures in the transmission process: hand washing (allows the decontamination of HCWs), staff cohorting (reduces patients mixing with contaminated HCWs), antibiotic restriction (reduces the probability of CPKP colonization per contact with contaminated HCW), screening and isolation of colonized admissions.

## Methods

### Ethics Statement

The study was approved by the Institutional Review Board of Laikon General Hospital. Informed consent was waived as all data were used anonymously.

### Setting and Data Collection

A prospective non-interventional study was conducted between May 2009 and June 2010 in a 30-bed surgical unit of a tertiary-care hospital located in Athens, Greece. The unit consisted of nine rooms; six rooms with three beds in each room and three rooms with four beds. The mean nurse to patient ratio was 1∶16. Hand-washing basins and dispensers containing alcohol-based disinfectants were located near all beds. All patients admitted to the unit were recorded in the database along with admission and discharge dates, time periods spent outside the unit, date and type of surgery.

The contact rate between patients and healthcare workers (HCWs) was estimated by direct observation of patients for periods of one hour. A total of 42 hours of observation, i.e. 14 hours per shift (06.00–15.00, 15.00–23.00 and 23.00–06.00), was attained. Contacts were defined as any contact of HCWs with the patient or the patient's surroundings.

Hand hygiene compliance was estimated by direct unobtrusive observation of patient-HCWs contacts. Observations of 20–30 minutes each were performed by a trained and validated observer who recorded health care activities. More specifically, during the observation-sessions, the observer recorded the opportunities for hand hygiene and the action performed by the HCW as either action performed (rubbing with an alcohol-based hand rub, washing with soap and water, both washing and rubbing) or not performed, according to the “My five moments for hand hygiene” WHO Hand Hygiene Improvement Strategy [26], [27]. Each opportunity corresponds to a hand hygiene action, performed or not. Thus, compliance was estimated as the ratio of the number of performed actions to the number of opportunities. Two-hundred and thirty opportunities were observed to estimate compliance.

**Table 1:** Parameter estimates used in the model of CPKP transmission.

Parameter	Symbol	Value used inthe model	Note
Number of beds	B	30	
Number of HCWs		24	Total daily number
Number of nursing staff		10	Total daily number
Discharge rate for uncolonized patients (/day)	*μ_μ_*	1/10.3	1/duration of stay of uncolonized patients
Discharge rate for colonized patients (/day)	*μ_c_*	1/22.9	1/duration of stay of colonized patients
Admission rate (/day)	*λ*	5.0–8.7	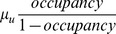 using monthly estimates of bed occupancy
Colonization prevalence on admission (%)	*φ*	0%–4.9%	Monthly estimates
Per capita contact rate (/patient/HCW/day)	*α*	1.4	
Probability of a patient becoming colonizedduring contact with contaminated HCW	*b_p_*	–	Estimated by the model
Probability of a HCW becoming contaminated during contact with colonized patient	*b_h_*	21.4%	CPKP was isolated from the hands of HCWs in 15 out of 70 contacts with colonized patients
Decontamination rate of HCWs (/day)	*μ_h_*	24	1/duration of contamination where duration is assumed 1 hour (1hour = 1/24 days)
Hand washing compliance	*p*	21%	

### Laboratory Data

Surveillance cultures (pharynx, rectal and from other sites, when clinically indicated) were obtained from all patients admitted to the surgical unit within 48 hours upon admission and every 7 days afterwards, until hospital discharge. The samples were inoculated on McConkey agar plates (OXOID) containing 0.5 mg/L of meropenem and incubated at 37°C for 48 hours. Colonies obtained on the meropenem-containing MacConkey agar plates that were visualized macroscopically as members of the *Enterobacteriaceae* were identified to the species level by the API 20E (bioMérieux, Marcy l’
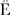
toile, France). All isolates identified as *K. pneumoniae* were examined for production of carbapenemases by combined disk synergy test utilizing disks containing meropenem, meropenem/EDTA, meropenem/boronic acid, and meropenem/EDTA+boronic acid as described previously [28]. The presence of *bla*_VIM_, *bla*_KPC_ genes were detected by polymerase chain reaction using consensus primers [29].

In order to estimate the probability that the hands of a HCW become contaminated after the contact with a CPKP colonized patient, HCWs who were aware of being observed, were asked to examine a patient known to be colonized with CPKP for a minimum of 30 seconds. After this procedure, their hands were cultured according to “the sterile bag” technique. The sampling solution contained neutralizers for antiseptics [30].

### Preliminary Evaluation of Transmission within the Unit

A preliminary evaluation of the main route of CPKP acquisition within the unit (colonized admissions and endogenous or environmental acquisition vs. cross-transmission) was performed by calculating the dispersion, i.e. the ratio variance/mean of the distribution of the number of colonized patients per day. The dependence created by cross-transmission leads to overdispersion in the number of colonized patients per day [31]. Thus, the distribution of the number of patients colonized at a given day is skewed and the variance to mean ratio of the number of patients colonized per day will exceed 1. Conversely, dispersion lower than 1 indicates that colonisations mainly result from endogenous or environmental acquisition and colonized admissions.

**Figure 2:**
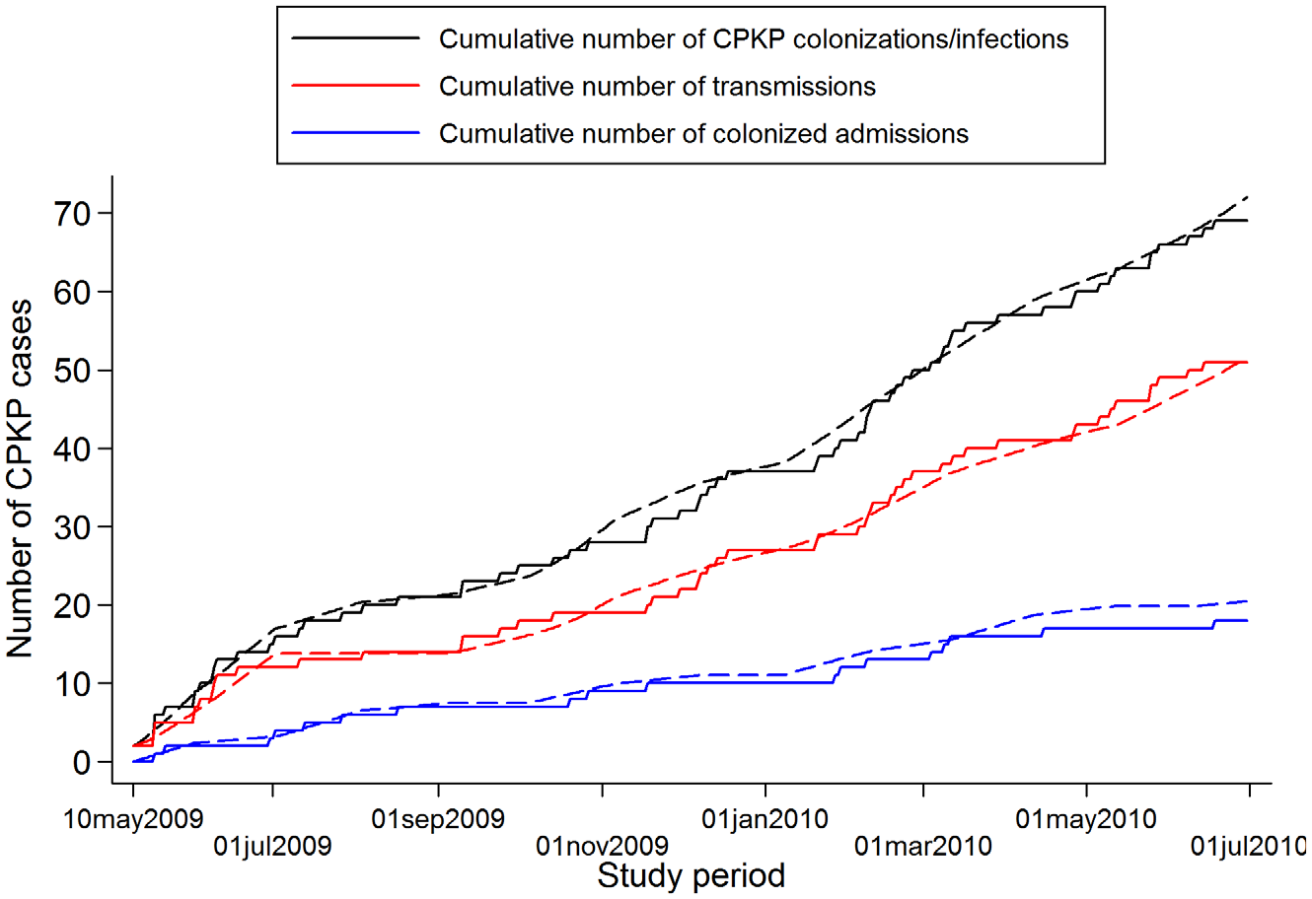
Observed number of patients with CPKP and model fit. Observed cumulative number of CPKP colonized patients (admissions and colonizations within the unit), CPKP colorizations within the unit and CPKP colonized admissions (solid lines) along with the corresponding model fit (dashed lines).

### Mathematical Model in the Absence of Infection Control Measures

The model that was used to describe the dynamics of CPKP is the Ross-Macdonald model for vector-borne diseases where health-care workers are the vectors transmitting CPKP from patient to patient [13], [14] [32]. The model assumes that the environment does not contribute to the transmission dynamics of CPKP. It consists of four differential equations describing the change in the number of the four different populations (Figure 1A), i.e. of uncolonized patients (X_p_), colonized patients (Y_p_), CPKP-free HCWs (X_h_) and contaminated HCWs (Y_h_):


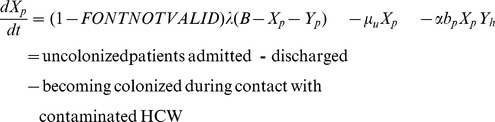



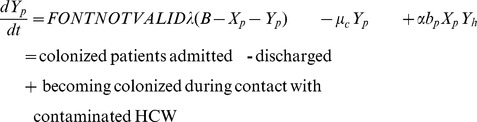



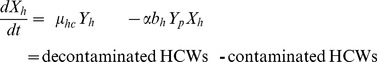



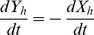


Patients are admitted to the ward at a rate λ per day and a fraction φ of them is already colonized with CPKP (B: the number of beds). They are discharged at a rate µ_u_ equal to 1/length of stay for non-colonized patients or at a rate µ_c_ equal to 1/length of stay for colonized patients. Patients mix with HCW at a per capita contact rate α contacts/patient per HCW per day and have a probability *b_p_* of becoming colonized during contact with a contaminated HCW. Once patients are colonized, they are assumed to remain colonized for the remainder of their stay in the ward.

HCWs become contaminated with probability *b_h_* per contact with a colonized patient and become decontaminated at a rate µ_h_ equal to 1/duration of contamination (the duration of contamination is usually assumed to be approximately 1 hour).

Fitting the model to the data allows obtaining an estimate of the basic reproduction number (R_0_), i.e. of the average number of secondary cases generated by a primary case (in the absence of infection control measures). For an epidemic to occur, more than one secondary cases has to be generated by the primary case, thus R_0_>1. In the case of vector borne transmission, R_0_ is the product of factors involved in the transmission from a colonized patient to a CPKP-free HCW (R_p_) and from a contaminated HCW to a susceptible patient (R_h_) and is given by: 
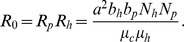


### Mathematical Model under Infection Control Measures

In the presence of infection control measures, it is of interest to estimate the effective reproduction number *R*, i.e. the actual average number of secondary cases produced by a primary case in the presence of e.g. hand hygiene measures, screening on admission for CPKP colonization and other control strategies. *R* is thus anticipated to be lower than *R_0_* and strategies are considered successful if they reduce it below unity.

In the presence of hand disinfection, the probability that a HCW will become contaminated per contact with a colonized patient (*b_h_*) is reduced by *p*%, where *p* denotes the hand hygiene compliance rate in the surgical unit. The effective reproduction number *R(p)* is then equal to (1−*p*)*R_0_*. Using this formula, the threshold compliance for control of transmission, i.e. for achieving *R(p)*<1, is *p*>1−1/*R_0_*. The effect of hand hygiene compliance (Figure 1B) can be incorporated in the model as follows [14]:


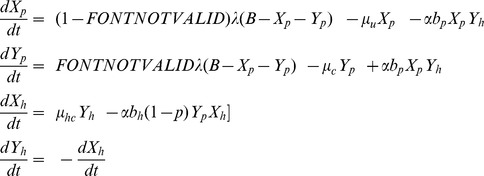


The model can be easily modified to account for the effect of additional control strategies. We further considered active surveillance, i.e. testing of patients on admission to detect CPKP carriers. Prompt identification enables healthcare providers to initiate proper interventions immediately, such as isolation of colonized or infected individuals, strict enforcement of appropriate barrier precautions and hand hygiene promotion, to minimize the opportunity for further CPKP transmission. This strategy can be modelled through a reduction of the parameter *φ* which denotes CPKP colonization prevalence on admission (Figure 1B). Another feature that can be taken into account is the effect of antibiotics on colonization (Figure 1B). Antibiotics may provide CPKP with a selective growth advantage that will result in higher probability of colonization. Thus, the probability *b_p_* of a patient being colonized per contact with a contaminated HCW can be decomposed into the product of a baseline probability *b_p0_* and a factor *f* representing the impact of antibiotics. If *r* is the relative risk of colonization associated with these agents and patients receive them for a fraction *d* of their stay in the unit, then *f = 1+d(r−1)* [13]. Antibiotic restriction policies could target to reducing the duration of administration of these agents from *d* to *d′*. In that case, *R_0_* is anticipated to decrease by *(d−d′)(r−1)/(1+d(r−1))*.

### Parameters and Model Fit

All of the parameters required for the model were obtained from data collection during the study with the exception of the probability of colonization *b_p_*. The model was simulated stochastically assuming Poisson rates over small time steps for each of the seven events included in the model (uncolonized admissions, uncolonized discharges, colonized admissions, colonized discharges, colonization of patients within the unit, contamination of HCWs, decontamination of HCWs). Overall, 1,000 simulations of the model were performed and the model was fit to the cumulative number of CPKP cases over time. The fit to the data allowed estimating the probability of colonization *b_p_*, the effective reproductive number *R* and the basic reproduction number *R_0_*. The model was fit using the Berkeley Madonna software.

**Figure 3:**
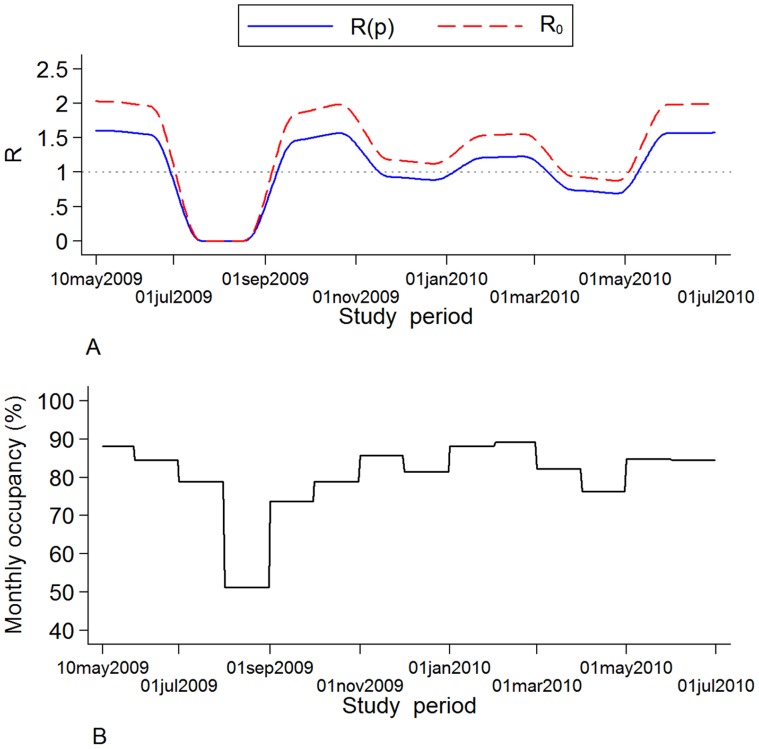
Bimonthly predicted effective reproduction number *R(p)* and observed occupancy within the surgical unit. A. Bimonthly predicted effective reproduction number *R(p)* (under the observed hand hygiene compliance rate of *p* = 21% during the study period) and the corresponding basic reproduction number *R_0_*. Values of *R(p)*>1 (dotted line) indicate the potential for an epidemic to occur. **B.** Observed occupancy within the surgical unit during the study period (monthly estimates).

### Simulations of the Impact of Control Efforts on the Prevalence of CPKP Colonization within the Unit

The transmission of CPKP was simulated within the surgical unit assuming an *R_0_* equal to 2. Other model parameters were set equal to their average values as obtained throughout the study period. We simulated the impact of control measures on colonization prevalence in the unit under two scenarios: endemic and hyperendemic setting.

In the endemic scenario, it was assumed that one CPKP colonized patient enters the surgical unit on day 0 and during the first 30 days the only infection control measure applied is hand hygiene with compliance *p* equal to 21%. After day 30 where colonization prevalence has increased to 8%–9%, additional infection control measures were implemented (improved hand hygiene compliance, active surveillance, antibiotic restriction policies). The impact of active surveillance was evaluated assuming 60% or 90% reduction in colonization prevalence on admission (*φ*). We evaluated the impact of attaining 50% reduction in the duration of antibiotic usage during patients' stay in the unit, assuming a relative risk *r* associated with antibiotic use equal to 3 [7], [10]. We further examined what would be anticipated if after 3 months of enhanced infection control measures, a more relaxed strategy consisting of enhanced hand hygiene compliance (60%) was adopted.

In the hyperendemic scenario, a high colonization prevalence of 20% was assumed on day 0. This estimate was obtained from unpublished data from another unit in the same hospital before an aggressive control strategy was implemented. In the simulations, infection control measures started on day 0 and the attained reduction in colonization prevalence over time was simulated.

## Results

### Estimates for the Model Parameters Obtained through Data Collection

During the study period, there were 850 admissions. The monthly daily bed occupancy ranged from 51.3% in August 2009 up to 89.2% in February 2010. The daily number of HCWs was on average 24, ten of which were nursing staff. A total of 69 patients were CPKP colonized; 18 were colonized on admission and the remaining 51 acquired CPKP after admission. During the study period, the monthly prevalence of CPKP colonization on admission (*φ*) varied from 0% to 4.9% with a median of 2.0%. The mean duration of stay in the unit was 10.3 days for non-colonized and 22.9 days for colonized patients. The per-capita contact rate 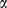 was estimated to be 1.4 contacts per patient per HCW per day. CPKP was isolated from the hands of HCWs in 15 (21.4%) out of a total of 70 contacts between HCWs and colonized patients. The observed hand hygiene compliance rate was 21%. Antibiotics use was evaluated in detail in a subsample of patients. Sixty-six percent of the patients received antibiotics during their stay. The most frequently administered antibiotics were second-generation cephalosporins (35%), metronizadole (21%), β-lactam/inhibitor combinations (16%) and fluoroquinolones (8%). Patients received antibiotics for a 33% of their stay (median estimate assuming duration of 0 days for those who did not receive antibiotics). The parameters used in the model are summarized in Table 1.

**Figure 4:**
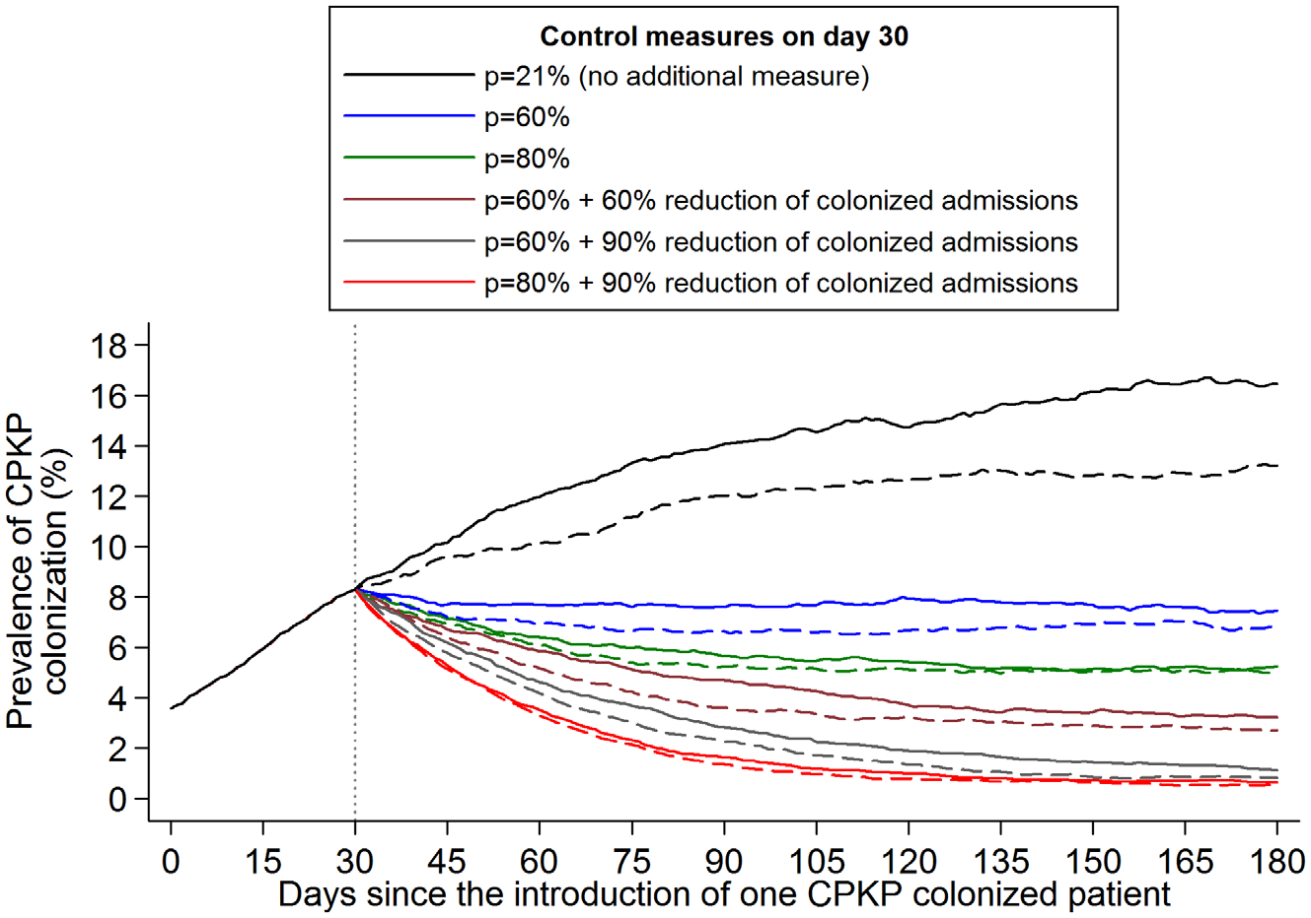
Impact of infection control measures on the prevalence of CPKP colonization in an endemic setting. One CPKP colonized patient enters the surgical unit on day 0 and the only infection control measure applied during the first 30 days is hand hygiene compliance (*p* = 21%). The impact of various infection control strategies implemented since day 30 on CPKP colonization prevalence was simulated (the mean of 1,000 simulations is shown). The evaluated scenarios include: **1.** adopt no additional infection control measures (hand hygiene compliance *p* = 21%), **2.** increase *p* to 60%, **3.** increase *p* to 80%, **4.** increase *p* to 60% and reduce colonization prevalence on admission of CPKP by 60% (through active surveillance and subsequent isolation or strict contact precautions for positive patients), **5.** increase *p* to 60% and reduce colonization prevalence on admission of CPKP by 90%, **6.** increase *p* to 80% and reduce colonization prevalence on admission of CPKP by 90%. Dashed lines (**- - -**) correspond to the above scenarios with the addition of 50% reduction in the duration of antibiotic usage during patients' stay in the unit (assuming a relative risk associated with antibiotic use equal to 3).

### Evaluation of Transmission within the Unit

The mean number of colonized patients per day was 2.05 and the variance 2.46 (Figure S1). Thus, dispersion was 1.20, indicating that cross-transmission was the main route of CPKP acquisition within the unit.

The model that was fitted to the cumulative number of CPKP cases over bimonthly intervals predicted satisfactorily the cumulative number of both colonized admissions and transmissions occurring within the surgical unit (Figure 2). The obtained estimates of *R_0_* and *R(p)* during the study period are shown in Figure 3A. With the exception of the summer months (July-August 2009) and of April 2010, *R_0_* was consistently higher than 1 suggesting that, in the absence of infection control measures, CPKP could be transmitted efficiently within the setting of the surgical unit. In the peak months, *R_0_* was approximately 2 indicating that, in the absence of any control measure, a colonized patient may generate 2 secondary cases on average. Using the observed hand hygiene compliance and the estimated *R_0_*, the effective reproduction number *R(p)* was also calculated. For long intervals during the study period, *R(p)* was higher than 1, indicating that the currently employed hand hygiene compliance rates were not adequate to inhibit CPKP transmission within the unit. The lowest values of *R_0_* and *R(p)* coincided with periods of low occupancy (Figure 3B).

**Figure 5:**
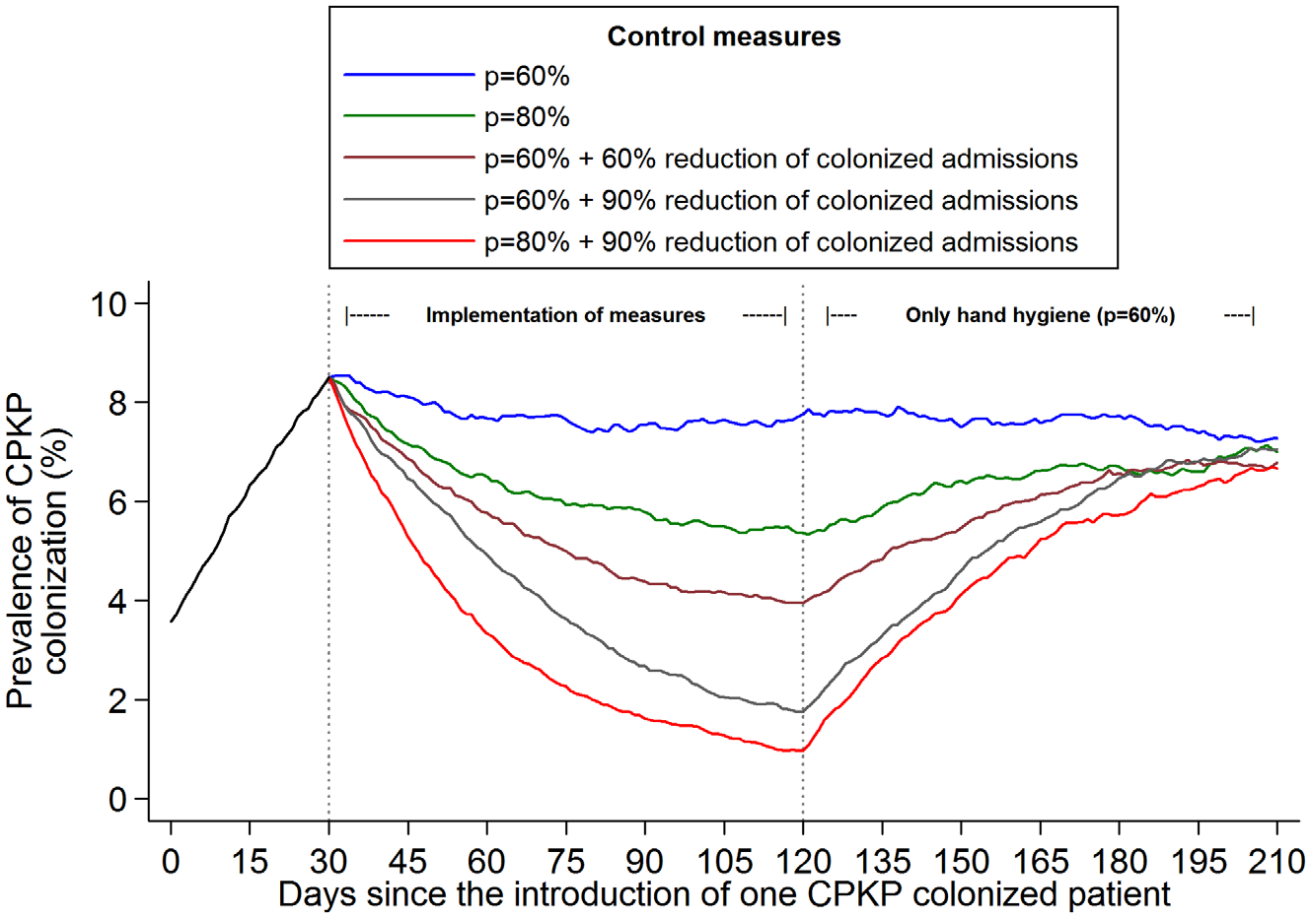
Impact of relaxing the infection control measures on the prevalence of CPKP colonization in an endemic setting. One CPKP colonized patient enters the surgical unit on day 0 and the only infection control measure applied during the first 30 days is hand hygiene compliance (*p* = 21%). Infection control measures are implemented during three months (day 30 - day 120). After day 120, only hand hygiene measures with 60% compliance are implemented. The evaluated scenarios during day 30-day 120 include: **1**. Hand hygiene compliance *p* = 60%, **2.** *p = *80%, **3.** *p* = 60% and reduce colonization prevalence on admission of CPKP by 60% (through active surveillance and subsequent isolation or strict contact precautions for positive patients), **4.** *p* = 60% and reduce colonization prevalence on admission of CPKP by 90%, **5.** *p* = 80% and reduce colonization prevalence on admission of CPKP by 90%.

### Infection Control Scenarios

The estimation of *R_0_* allowed the determination of the threshold for hand hygiene compliance in order to eradicate CPKP from the unit, in the absence of further colonized admissions. Thus, it was estimated that in periods where *R_0_* is 2, hand hygiene compliance should exceed 50% in order to attain an effective reproduction number below unity. An additional policy of reducing the duration of antibiotic use by 20% or 40% had an impact illustrated in Figure S2. In the presence of these antibiotic restriction policies, the threshold of hand hygiene compliance for *R(p)<*1 is reduced to 45.6% and 40.5%, respectively.

**Figure 6:**
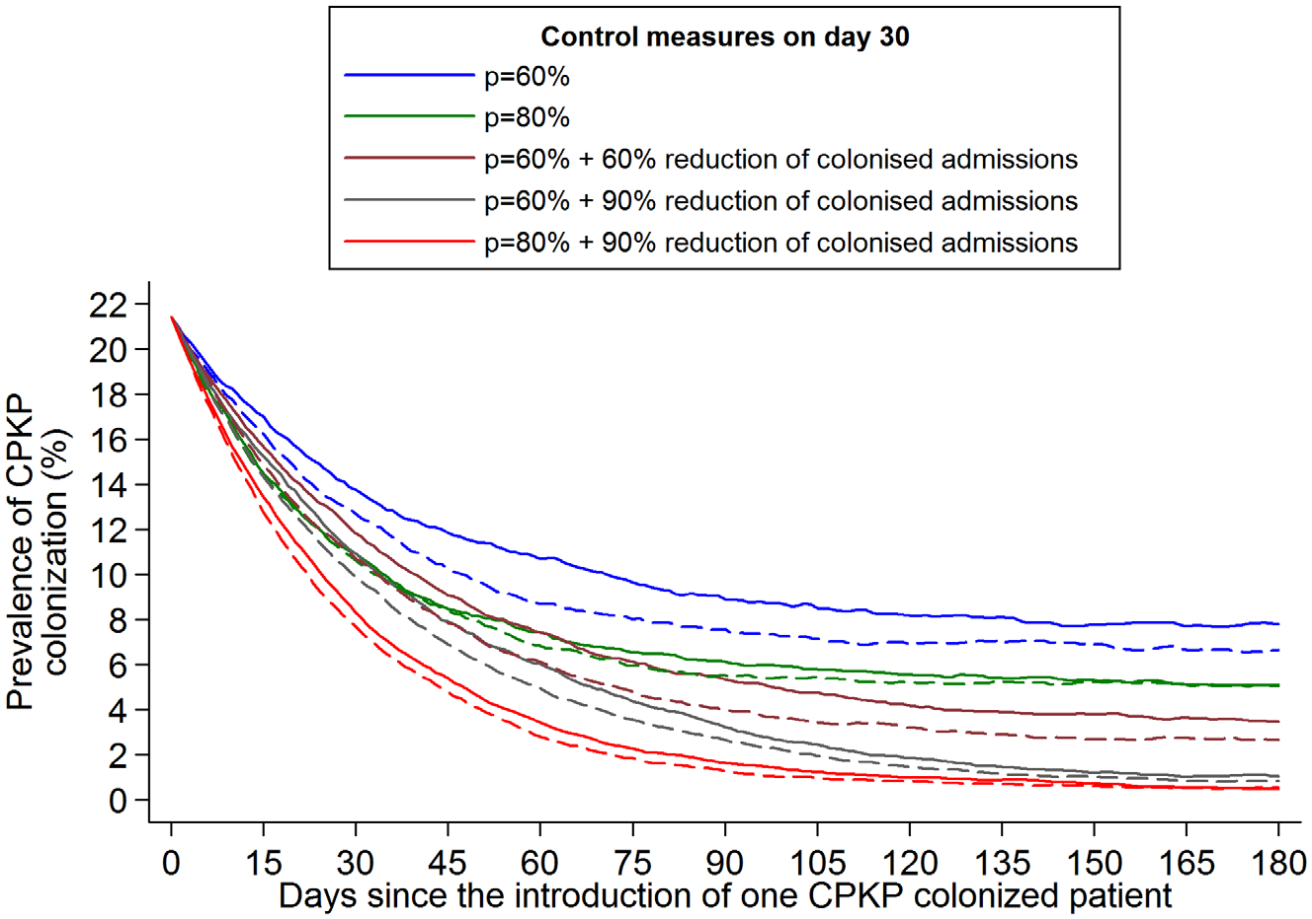
Impact of infection control measures on the prevalence of CPKP colonization in an hyperendemic setting. Infection control measures start on day 0 where a high colonization prevalence of 21% was assumed. The evaluated scenarios include: **1**. Hand hygiene compliance *p* = 60%, **2.** *p = *80%, **3.** *p* = 60% and reduce colonization prevalence on admission of CPKP by 60% (through active surveillance and subsequent isolation or strict contact precautions for positive patients), **4.** *p* = 60% and reduce colonization prevalence on admission of CPKP by 90%, **5.** *p* = 80% and reduce colonization prevalence on admission of CPKP by 90%. Dashed lines (**- - -**) correspond to the above scenarios with the addition of 50% reduction in the duration of antibiotic usage during patients' stay in the unit (assuming a relative risk associated with antibiotic use equal to 3).

The transmission of CPKP was simulated within the surgical unit assuming that one CPKP colonized patient enters the surgical unit on day 0 and during the first 30 days the only infection control measure applied is hand hygiene with compliance *p* equal to 21%. After day 30, infection control measures are implemented. The effect of different scenarios on the prevalence of CPKP colonization within the unit is shown in Figure 4. Hand hygiene compliance rates of 60% may contain transmission but this measure alone did not have a substantial impact on further reducing CPKP prevalence as there was ongoing influx of colonized patients. A decrease in the colonization prevalence within the unit was estimated only when improvement in hand hygiene compliance was coupled with measures that would lead to a decrease in the number of colonized admissions (Figure 4). Such measures could be CPKP screening and isolation/cohorting of colonized admissions along with contact precautions. Under an aggressive control strategy (60%–80% hand hygiene compliance and 60%–90% reduction in the number of colonized admission), CPKP prevalence would fall below the baseline levels (day 0) within 1–3 months of implementation. In this case, antibiotics restrictions did not have a substantial additional benefit. However, measures leading to 50% reduction in the duration of antibiotics usage were predicted to have an impact when coupled with low or moderate efficacy measures such as e.g. hand hygiene compliance of 21%.

We further examined what would be anticipated if after 3 months of enhanced infection control measures (days 30–120), a more relaxed strategy consisting of enhanced hand hygiene compliance (60%) was adopted (Figure 5). Under the evaluated scenarios of 80% hand hygiene compliance or combined 60%–80% hand hygiene compliance and 60%–90% reduction in the number of colonized admission, CPKP prevalence levels were predicted to start increasing. After approximately three months, i.e. by day 210, these levels converged to the levels achieved by the 60% hand hygiene compliance strategy.

An additional scenario that was explored was the impact of these measures in a hyperendemic setting. Measures were implemented on day 0 where a high colonization prevalence of 20% was assumed (Figure 6). A rapid decline was predicted under all evaluated infection control strategies. A plateau in the decline was observed approximately 4 months (day 120) after the implementation of measures. The reduction on day 120 compared to day 0 ranged between 62%–76% for the scenarios of 60%–80% hand hygiene compliance, with or without antibiotics restrictions, and between 80%–97% for the scenarios of aggressive measures (60%–80% hand hygiene compliance and 60%–90% reduction in the number of colonized admission with or without antibiotics restrictions).

## Discussion

The present study provides important information on the estimates of CPKP transmissibility in a surgical unit and on the impact of various interventions for successful containment. By using data on the prevalence of CPKP on admission as well as on the occurrence of new acquisition within the unit, it was estimated that the basic reproduction number *R_0_* of CPKP exceeded 1 and reached approximately 2 in the peak months. In time periods when the basic reproductive number was 2, admission of a single CPKP carrier would, on average, have generated 2 new cases. These findings indicate that CPKP has the potential to spread and trigger outbreaks in the healthcare setting.

The *R_0_* estimates for CPKP in the surgical unit were lower than the *R_0_* predicted by similar modeling studies for VRE (*R_0_* = 3.81) and MRSA (*R_0_* = 10) that were conducted in ICUs [13], [14]. Variations in *R_0_* for these pathogens may represent differences in the transmission dynamics of the organisms per se or they may reflect differences in the hospital settings where the studies were conducted; ICU, non-ICU, HCW to patient ratios and density of patient population. Indeed, the latter appears to play an important role in cross-transmission of CPKP in our hospital. As was shown above, *R_0_* estimates for this pathogen paralleled the fluctuations in bed occupancy, i.e. the higher the level of bed occupancy, the higher the *R_0._* Similarly, Grundmann et al [14] reported that clustered cases of MRSA within an ICU occurred more often during periods of staff deficit when the patient to nurse ratio was higher.

Under the recorded infection control practices in the surgical unit (21% hand hygiene compliance), the effective reproductive number for CPKP exceeded unity for long periods of time. Apparently, the compliance level with hand hygiene was not adequate to contain cross-transmission within the unit. In the absence of additional infection control measures and in time periods when the estimated *R_0_* was 2, the minimum compliance level with hand hygiene necessary to control transmission was estimated to be 50%. However, in settings with similarly low hand hygiene compliance rates, it is questionable whether a substantial improvement can be achieved, and if so, whether such an improvement could be sustained over time. Furthermore, the simulated impact of hand disinfection alone on the prevalence of CPKP colonization, even at high compliance rates, was poor, given the constant influx of new colonized patients into the unit. It should be noted that the recorded monthly prevalence of CPKP colonization on admission was on average 2.0% and ranged between 0% and 4.9%. Thus, it is clear that additional measures should be employed concurrently with improvement in hand hygiene compliance in order to reduce the prevalence of CPKP in an endemic setting where constant importation of new cases occurs.

By applying the mathematical model on the antibiotic consumption data, it was found that 40% reduction in antibiotic use could reduce the threshold of hand hygiene compliance from 50% to 40% in order to control CPKP cross-transmission in the unit. These results, in conjunction with previous studies that have shown that the intensive use of antibiotics has been associated with a high probability of CPKP colonization [33], [34], indicate that antibiotic restriction policies could have some effect on new acquisition of CPKP. However, as was presented in a recent review on antimicrobial stewardship, the reductions in antibiotic use that could be achieved were less than 38% and improvements in antimicrobial resistance rates were observed 6 months after interventions [35].

In addition to the interactions between hand hygiene and antibiotic restriction for CPKP containment, we have also evaluated the impact of different scenarios involving hand hygiene at various compliance rates in conjunction with reduction in the influx of new colonized patients. The latter could have been achieved by active surveillance of all new admissions for CPKP carriage coupled with isolation or cohorting of all carriers along with strict contact precautions. In these scenarios, it was predicted that 60% to 90% reduction in colonized admissions in conjunction with improvement in hand hygiene compliance up to 60%, would result in rapid decline in CPKP prevalence in an endemic as well as in a hyperendemic setting. It is important to note, however, that as soon as these measures were replaced by more relaxed infection control practices, the benefit in reducing CPKP prevalence could be vanished within approximately 3 months. In addition to isolation/cohorting of all CPKP carriers, assigning dedicated staff to carriers has been shown to be a successful way to halt intra-hospital transmission [36], [37]. This strategy, however, cannot be easily implemented and sustained, particularly in facilities with limited resources.

The present study is one of the few studies that employed mathematical modeling on surveillance data in order to estimate the basic reproduction number of a nosocomial pathogen and to assess the impact of various infection control strategies on its transmission dynamics. Furthermore, it is unique in that it provides these estimates for CPKP, an emerging public health threat. However, the findings of this report are subject to several limitations. First, the model assumes that transmission occurs exclusively through the HCWs, not taking into account possible transmission through the inanimate environment. This is a common assumption in similar studies [13]–[15]. However, data suggest that the environment plays a minimal role for the spread of *Enterobacteraceae* in a hospital setting [38]. Second, in modeling hand hygiene compliance, it was assumed that the efficacy of the hand cleansing process was 100%. This is a common assumption is similar models of hospital transmission [13]–[15]. Third, it should be noted that, in Greek hospitals, patients' relatives and visitors remain within the rooms during relatively long periods during the day and may contribute to cross-transmission. This effect, however, was not modeled, as additional data for visitors, similar to those collected for the HCWs, were not available (contact rate, probability of becoming contaminated during contact etc). However, the visitors’ effect is anticipated to be minimal since they do not systematically touch or care other patients.

In conclusion, the findings presented herein have important implications in designing infection control strategies to contain or even eliminate CPKP. The estimates for CPKP transmissibility ascertain that this pathogen, in the absence of adequate infection control practices, can spread and persist within the hospital setting very efficiently. In healthcare facilities where CPKP endemicity is sustained by cross-transmission as well as by the influx of already colonized patients, it is imperative that control policies should target both these mechanisms. The use of surveillance culture on admission and subsequent separation of carriers from non-carriers coupled with improved hand hygiene compliance and contact precautions may attain maximum containment of CPKP in endemic and hyperendemic settings.

## Supporting Information

Figure S1 **Distribution of the number of colonized patients per day.** The dispersion parameter (variance/mean) is 1.20 indicating that cross-transmission is the main route of CPKP acquisition within the unit.
(TIF)

Figure S2 **Prediction of the effective reproduction number** ***R(p)*** **of CPKP at different levels of hand washing compliance (** ***p*** **) assuming an** ***R_0_*** **of 2.** Values of *R(p)>*1 (i.e. above the dotted line) indicate the potential for an epidemic to occur. The threshold hand hygiene compliance for *R(p)<*1 is 50% (black line). The red and blue lines depict *R(p)* at different levels of hand washing compliance under a 20% or 40% reduction, respectively, in the duration of antibiotic usage during patients' stay in the unit (assuming a relative risk associated with antibiotic use equal to 3). In the presence of these antibiotic restriction policies, the threshold hand hygiene compliance for *R(p)<*1 is estimated 45.6% and 40.5%, respectively.
(TIF)
