# Supplementary figures and images for: Transmission Dynamics of Carbapenemase-Producing Klebsiella Pneumoniae and Anticipated Impact of Infection Control Strategies in a Surgical Unit
Source: PLoS One. 2012 Jul 31;7(7):e41068. doi: 10.1371/journal.pone.0041068 (PMC3409200; doi:10.1371/journal.pone.0041068)

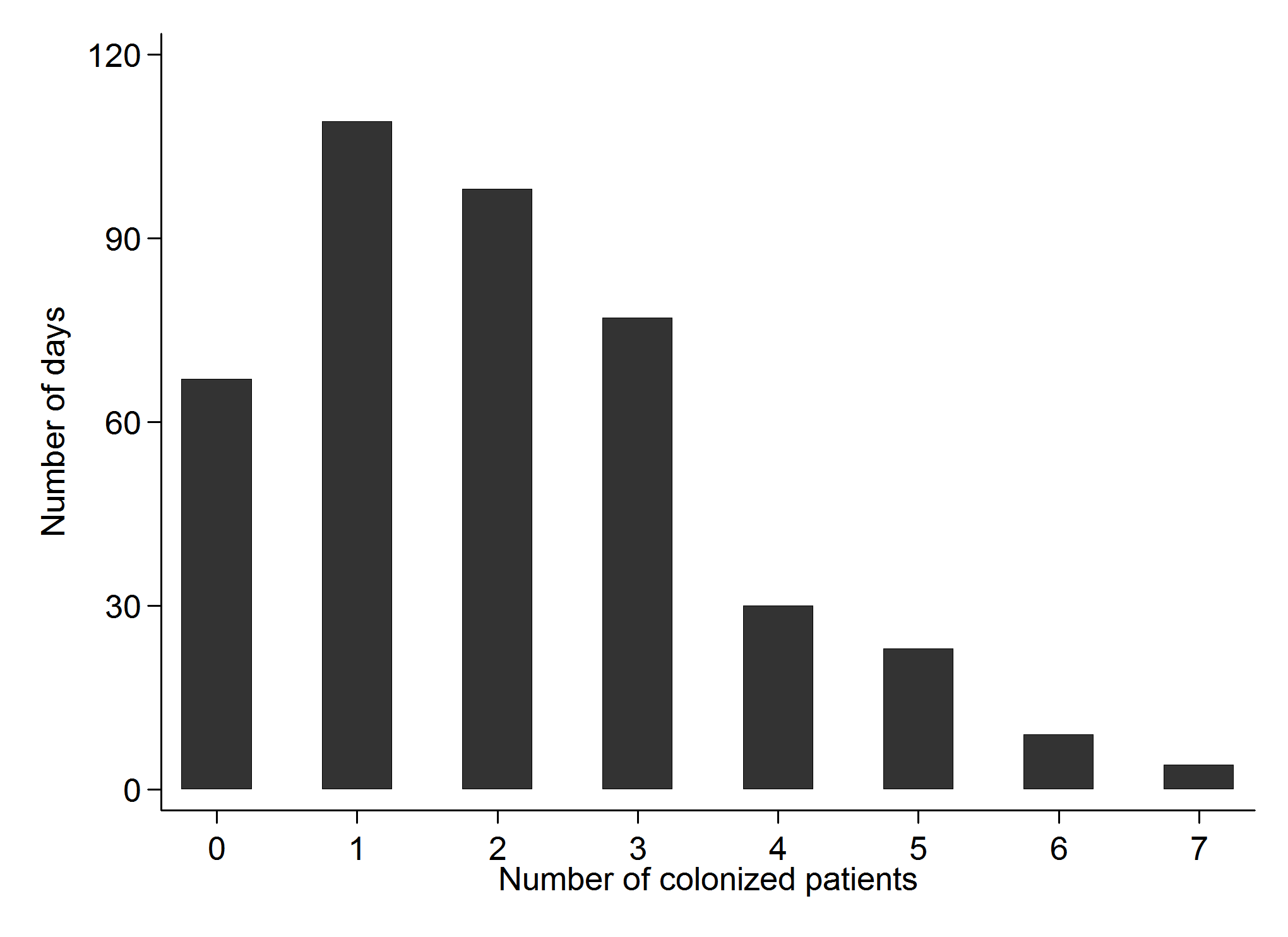

Supplement: Figure S1 — Distribution of the number of colonized patients per day. The dispersion parameter (variance/mean) is 1.20 indicating that cross-transmission is the main route of CPKP acquisition within the unit. (TIF) [file pone.0041068.s001.tif]

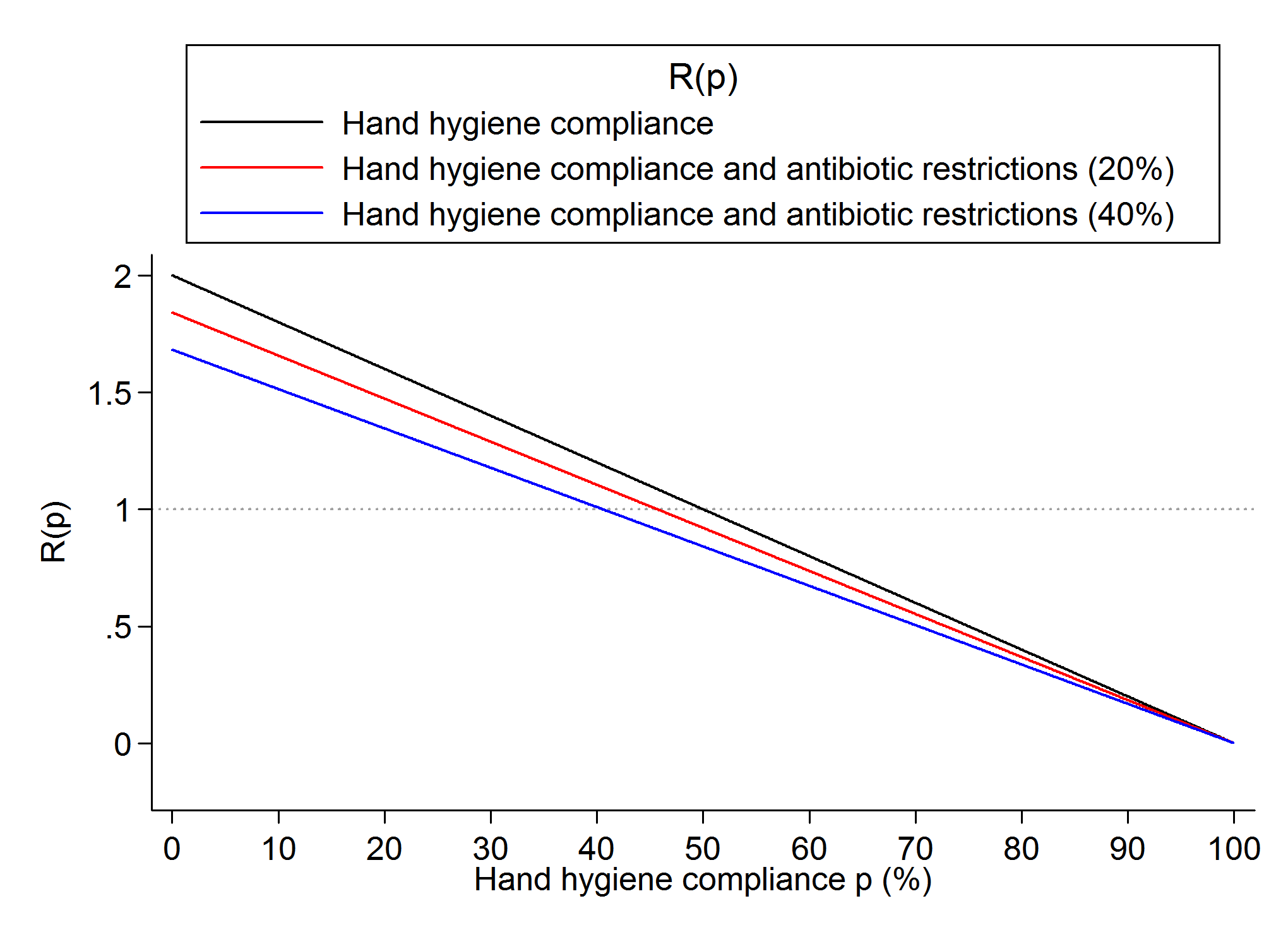

Supplement: Figure S2 — Prediction of the effective reproduction number R(p) of CPKP at different levels of hand washing compliance ( p ) assuming an R0 of 2. Values of R(p)>1 (i.e. above the dotted line) indicate the potential for an epidemic to occur. The threshold hand hygiene compliance for R(p)<1 is 50% (black line). The red and blue lines depict R(p) at different levels of hand washing compliance under a 20% or 40% reduction, respectively, in the duration of antibiotic usage during patients' stay in the unit (assuming a relative risk associated with antibiotic use equal to 3). In the presence of these antibiotic restriction policies, the threshold hand hygiene compliance for R(p)<1 is estimated 45.6% and 40.5%, respectively. (TIF) [file pone.0041068.s002.tif]
